# Relationship Between Weekly Patterns of Caloric Intake and Reported Weight Loss Outcomes: Retrospective Cohort Study

**DOI:** 10.2196/mhealth.8320

**Published:** 2018-04-16

**Authors:** Christine Hill, Brian W Weir, Laura W Fuentes, Alicia Garcia-Alvarez, Danya P Anouti, Lawrence J Cheskin

**Affiliations:** ^1^ Johns Hopkins Weight Management Center Johns Hopkins Bloomberg School of Public Health Johns Hopkins University Baltimore, MD United States; ^2^ Department of Health, Behavior and Society Johns Hopkins Bloomberg School of Public Health Johns Hopkins University Baltimore, MD United States; ^3^ Lerner Center for Public Health Promotion Johns Hopkins Bloomberg School of Public Health Johns Hopkins University Baltimore, MD United States

**Keywords:** mobile apps, weight reduction, caloric restriction, diet habits

## Abstract

**Background:**

Although millions of overweight and obese adults use mobile phone apps for weight loss, little is known about the predictors of success.

**Objective:**

The objective of this study was to understand the relationship between weight loss outcomes and weekly patterns of caloric intake among overweight and obese adults using a mobile phone app for weight loss.

**Methods:**

We examined the relationship between weekly patterns of caloric intake and weight loss outcomes among adults who began using a weight loss app in January 2016 and continued consistent use for at least 5 months (N=7007). Unadjusted and adjusted linear regression analyses were used to evaluate the predictors of percentage of bodyweight lost for women and men separately, including age, body mass index category, weight loss plan, and difference in daily calories consumed on weekend days (Saturday and Sunday) versus Monday.

**Results:**

In adjusted linear regression, percentage of bodyweight lost was significantly associated with age (for women), body mass index (for men), weight loss plan, and differences in daily caloric intake on Mondays versus weekend days. Compared with women consuming at least 500 calories more on weekend days than on Mondays, those who consumed 50 to 250 calories more on weekend days or those with balanced consumption (±50 calories) lost 1.64% more and 1.82% more bodyweight, respectively. Women consuming 250 to 500 calories or more than 500 calories more on Mondays than on weekend days lost 1.35% more and 3.58% more bodyweight, respectively. Compared with men consuming at least 500 calories more on weekend days than on Mondays, those consuming 250 to 500 calories or more than 500 calories more on Mondays than on weekend days lost 2.27% and 3.42% less bodyweight, respectively.

**Conclusions:**

Consistent caloric intake on weekend days and Mondays or consuming slightly fewer calories per day on Mondays versus weekend days was associated with more successful weight loss.

**Trial Registration:**

ClinicalTrials.gov NCT03136692; https://clinicaltrials.gov/ct2/show/NCT03136692 (Archived by WebCite at http://www.webcitation.org/6y9JvHya4)

## Introduction

### Weekly Patterns in General Health-Related Behaviors

Past research has demonstrated that behavioral differences between weekdays and weekends have a significant impact on health-related and dietary decisions. In particular, studies have found a common pattern of increased caloric intake and decreased physical activity on the weekend as compared with weekdays [1-4]. This circaseptan periodicity is not only limited to consumption but also seems to affect information-seeking behaviors. Google searches containing the word *healthy* are highest on Monday and Tuesday, as people remotivate themselves after the weekend, and thereafter, their motivation declines until rebounding again on Sunday [5]. Similar patterns have been detected in information-seeking behaviors specific to smoking cessation [6] and in some HIV-related behaviors [7]. This renewed interest in health at the start of the week is sometimes referred to as the “Monday effect” or the “Monday phenomenon” [8,9].

### Weekly Patterns in Weight Loss Behaviors

Previous research has demonstrated that these weekly intake patterns may contribute to a slower rate of weight loss due to cessation of weight loss on weekend days as well as long-term weight gain [10]. However, other research suggests that fluctuating caloric intake across the week is not associated with weight gain over time. This is due to compensatory patterns of decreased caloric intake that often take place when the weekend ends. In one study, those that compensated the most for their weekend behavior during the week were the most likely to maintain their weight rather than gain [11]. A small trial (n=27) revealed that participants tended to gain weight during high-risk periods, such as weekends, but “weight losers” were more likely to compensate for this gain beginning on Mondays. Weight losers were also more likely to show a pattern of weekly weight gain and loss surrounding the weekend than nonlosers [12]. Whether or not cyclic patterns affect weight loss has not yet been clarified in a large sample of adults. Thus, we examined a large dataset from a mobile phone app to understand the influence of weekly patterns of caloric intake on weight loss success.

This study analyzes data logged by 7007 new users of *Lose It!*, a weight loss mobile phone app designed for easy caloric tracking [13]. *Lose It!* provides a free mobile and Web interface for users to construct a customized weight loss plan by entering their goal weight and desired weekly weight loss. Using the user’s height, current weight, gender, and age, the app generates a daily caloric budget designed for the user to reach their goal. Users can then log their caloric intake, exercise, and weight to track progress toward their goal. Our hypothesis was that users with inconsistent caloric intake on Mondays versus weekends would be less successful in their weight loss efforts.

## Methods

### Recruitment

FitNow Inc provided deidentified *Lose It!* data to researchers at the Johns Hopkins Bloomberg School of Public Health for analysis (ClinicalTrials.gov NCT03136692b). The dataset included users who actively used the app each month from January to May 2016. Specifically, the dataset was limited to users who logged food at least 8 times during the first or second half of each month (ie, January, February, March, April, and May). We further limited the sample to new users located in United States and Canada, between 18 and 80 years of age, and who are overweight (ie, 25<body mass index [BMI]<30) or obese (ie, BMI>30). The obtained data included: user ID number, sex, age, height, weight, number of times the user logged weight, number of days the user logged food, number of days the user logged exercise, number of food calories logged each day, number of exercise calories logged each day, daily caloric budget (for chosen weight loss plan), estimated energy requirement, and whether or not the user purchased the premium version of the app. Data cleaning consisted of eliminating duplicates and placing valid ranges on each variable.

**Figure 1:**
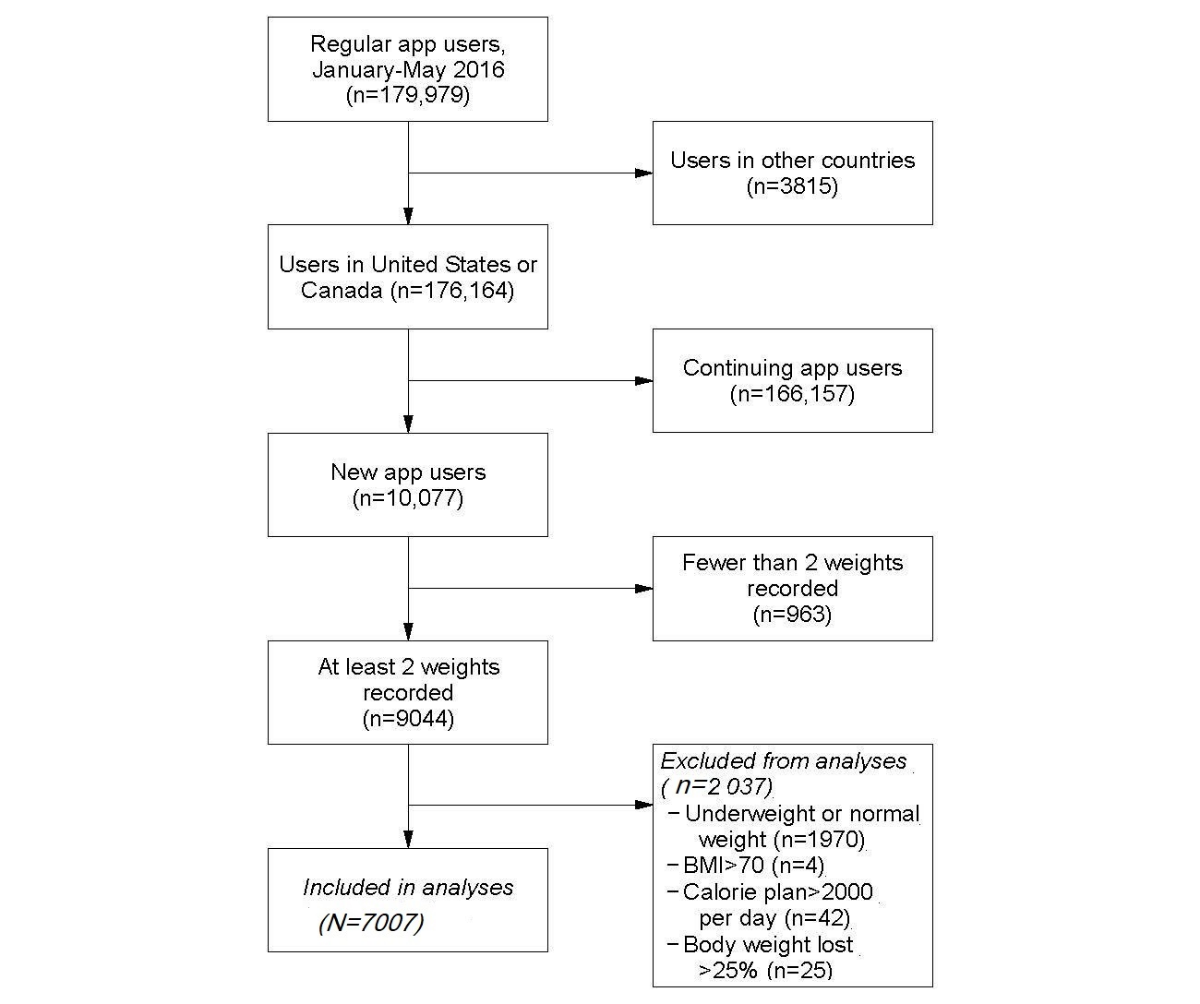
Inclusion of regular Lose It! app users between 18 and 80 years of age in analyses. Regular users are defined as users logging food at least 8 times during the first or second half of each month (January, February, March, April, and May). BMI: body mass index.

Among 176,164 individuals in the United States or Canada who were regular users of *Lose It!* from January through May 2016, we identified 10,007 as new users. Among them, 90.37% (9044/10,007) had at least two weigh-ins recorded, and 78.26% (7078/9044) of those were overweight or obese by BMI criteria. Finally, an additional 1.00% (71/7078) were excluded for either having a BMI greater than 70, having a weight loss plan with a caloric budget greater than 2000 calories per day, or reporting weight loss of more than 25% of starting bodyweight, yielding a final sample size of 7007 users (see Figure 1).

### Statistical Analysis

The primary outcome was the percentage of bodyweight lost over the 5-month window (January 2016 through May 2016) and was calculated by subtracting the final weight measurement from the first weight measurement and dividing the resulting value by the first weight measurement. The primary predictor of interest was the difference in reported calorie consumption between weekend days and Mondays, and this was calculated by subtracting the mean calories consumed on Mondays from the mean calories consumed on weekend days (Saturdays and Sundays). Thus, negative values indicated that more calories were consumed on Mondays than weekend days, whereas positive values indicated that fewer calories were consumed on Mondays than weekend days. This difference in calorie intake was then categorized into the following groups: less than −500 kcal, −500 kcal to −250 kcal, −250 kcal to −50 kcal, −50 kcal to 50 kcal, 50 kcal to 250 kcal, 250 kcal to 500 kcal, and more than 500 kcal. In regression analyses, additional covariates include years of age (ie, 18-24 years, 25-34 years, 35-44 years, 45-54 years, 55-64 years, and 65-80 years), sex, BMI category (ie, overweight, obesity I, obesity II, and extreme obesity), and user weight loss plan in pounds per week (<1 lb, ≥1 to <1.5 lb, ≥1.5 to <2 lb, and ≥2 to <4 lb). We did not include independent variables as continuous as many did not have linear relationships with the outcome variable, percent bodyweight lost. We categorized the predictors to allow non-linearity and for ease of interpretation.

Preliminary analyses described the distributions of mean daily calories consumed and calories consumed on Mondays relative to weekend days. Because women and men tend to differ in mean caloric intake [14], we presented descriptive data for women and men separately. We also estimated the associations between the predictor variables and the percentage of bodyweight lost for women and men. We performed two sets of linear regression of the percentage of weight loss. The first consisted of unadjusted regressions that included only one predictor (age, sex, initial BMI category, weight loss plan, or calories consumed on Mondays vs weekend days). Subsequently, an adjusted linear regression model was performed that included all of these predictors.

As we wanted to make statistical comparisons between the coefficient estimates for women and men, we estimated the coefficients in joint models. This approach produced coefficient estimates identical to running separate models for men and women, but allowed for direct statistical comparison of coefficients for men and women. All analyses were conducted using Stata/SE 14.2 (College Station, TX) [15]. All findings reported as “significant” have a *P* value of <.001, unless otherwise stated.

## Results

All US states, the District of Columbia, and all of the Canadian provinces were represented among the 7007 *Lose It!* users included in these analyses. Two-thirds (67.10%, 4702/7007) of users were identified as women, and the distributions of covariates for women and men and their relationships with mean daily calories were presented (see Table 1). The distributions of age, initial BMI category, weight loss plan, and daily calories consumed on Mondays relative to weekend days were significantly different between women and men. Women in the age groups of 18-24 years and 25-34 years tended to consume more similar amounts of calories on Mondays and weekend days than men did. Women were more likely to be overweight, whereas men were more likely to be in the lowest obesity category (Obesity I). Additionally, men were inclined to adopt more aggressive weight loss plans, as measured in pounds per week. Regarding the mobile phone app use, the mean number of recorded weigh-ins was 19.7 (SD 21.8, range 2-123).

**Table 1:** Individual characteristics and daily caloric intake for women and men using the *Lose It!* mobile phone app (N=7007). BMI: body mass index.

Characteristics		Distributions		Kcal per day	
		Women, n (%)	Men, n (%)	Women, mean (SD)	Men, mean (SD)
Total sample		4702 (100.00)	2305 (100.00)	1313 (302)	1737 (399)
**Age (years)**					
	18-24	643 (13.68)	217 (9.41)	1329 (311)	1727 (391)
	25-34	1110 (23.61)	486 (21.08)	1364 (308)	1841 (418)
	35-44	1119 (23.80)	588 (25.51)	1345 (315)	1802 (401)
	45-54	956 (20.33)	497 (21.56)	1284 (282)	1696 (395)
	55-64	620 (13.19)	347 (15.05)	1241 (275)	1647 (354)
	65-80	254 (5.40)	170 (7.38)	1196 (246)	1530 (302)
**BMI classification**					
	Overweight	2083 (44.30)	897 (38.92)	1275 (273)	1726 (358)
	Obesity I	1348 (28.67)	812 (35.23)	1308 (298)	1698 (393)
	Obesity II	715 (15.21)	344 (14.92)	1330 (311)	1769 (432)
	Extreme obesity	556 (11.82)	252 (10.93)	1447 (358)	1861 (477)
**Weight loss plan (lb/week)**					
	<1	157 (3.34)	91 (3.95)	1531 (328)	2022 (482)
	1 to <1.5	1405 (29.88)	538 (23.34)	1385 (292)	1829 (408)
	1.5 to <2	1318 (28.03)	507 (22.00)	1299 (275)	1749 (362)
	2 to <4	1822 (38.75)	1169 (50.72)	1249 (306)	1668 (384)
**Daily calories on Mondays versus weekend days**					
	More than 500 on weekend days	89 (1.89)	140 (6.07)	1489 (327)	1955 (389)
	More than 250 to 500 on weekend days	415 (8.83)	381 (16.53)	1418 (290)	1818 (408)
	More than 50 to 250 on weekend days	1617 (34.39)	742 (32.19)	1317 (299)	1696 (377)
	More than or less than 50 calories	1230 (26.16)	422 (18.31)	1267 (292)	1679 (388)
	More than 50 to 250 on Mondays	1029 (21.88)	447 (19.39)	1296 (300)	1703 (401)
	More than 250 to 500 on Mondays	204 (4.34)	129 (5.60)	1392 (309)	1785 (377)
	More than 500 on Mondays	40 (0.85)	33 (1.43)	1444 (300)	1945 (490)

**Figure 2:**
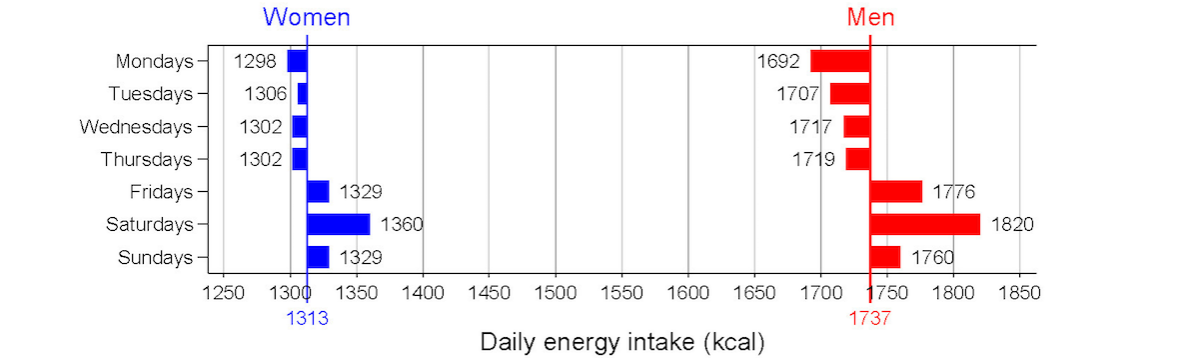
Caloric intake by day of the week relative to mean caloric intake for women (n=4702) and men (n=2305) who used the *Lose It!* mobile phone app.

**Figure 3:**
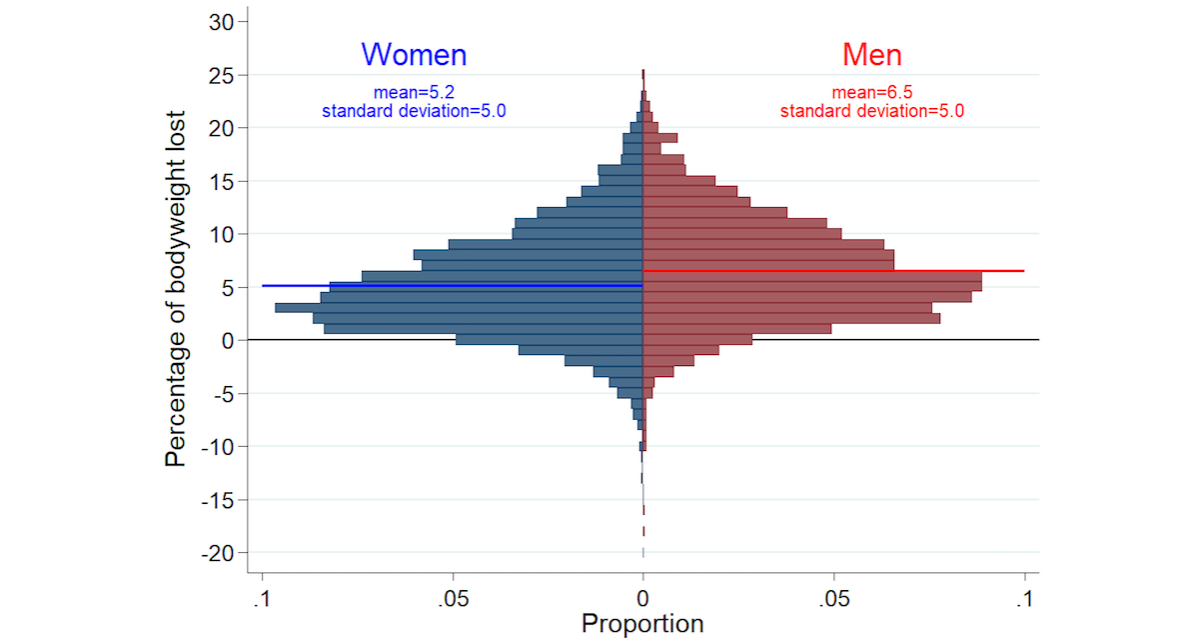
Distribution of percentage of bodyweight lost for women (n=4702) and men (n=2305) who used the *Lose It!* mobile phone app.

Women consumed significantly fewer calories per day than men (1313 vs 1737), but the relationships between individual characteristics and caloric intake were similar for women and men. For both, the highest mean caloric consumption was seen in those in the age group of 25-34 years, and caloric intake decreased in the older age groups. For both, mean caloric intake increased with initial BMI category, and decreased with the aggressiveness of the individual weight loss plan. For both, mean caloric intake was lower with similar caloric intake on Mondays and weekend days, and mean caloric intake increased as the imbalance between Mondays and weekend days increased. For both women and men, mean daily caloric intake was lowest on Mondays (1298 vs 1692) and highest on Saturdays (1360 vs 1820; see Figure 2). For women, compared with mean caloric intake on Mondays, caloric intake was within 8 calories on Tuesdays, Wednesdays, and Thursdays, was 31 calories higher on Fridays and Sundays, and was 62 calories higher on Saturdays. Men followed a similar, but more extreme, pattern: compared with mean caloric intake on Mondays, caloric intake was within 27 calories on Tuesdays, Wednesdays, and Thursdays, was 84 calories higher on Fridays, was 128 calories higher on Saturdays, and was 68 calories higher on Sundays. Figure 3 presents the distributions of percentage of bodyweight lost for women and men at the end of the 5-month period. Among women, 1.40% (66/4702) had no weight change, 10.80% (508/4702) gained weight, and 87.79% (4128/4702) lost weight, with a mean of 5.2 (SD 5.0) percent bodyweight lost. Among men, 0.78% (18/2305) had no weight change, 5.98% (138/2305) gained weight, and 93.18% (2148/2305) lost weight, with a mean 6.5 (SD 5.0) percent bodyweight lost.

In unadjusted analyses, all 4 predictors (age, BMI, weight loss plan, and daily calories on Mondays vs weekend days) were significantly associated with percentage of weight lost for both women and men (see Table 2). For women, percentage of weight lost was lower in the youngest (18-24 years) and oldest (65-80 years) age groups, and highest in the age group of 45-54 years. For men, percentage of weight lost was lowest in the age group of 65-80 years. For both women and men, weight loss was lowest among participants who were classified in the BMI category as overweight versus those classified as obese, and percentage of weight lost increased with more aggressive weight loss plans.

Compared with the women with caloric intake of more than 500 calories more on weekend days than Mondays, those consuming 50 to 250 more calories on weekend days relative to Mondays or similar calories on weekend days and Mondays (±50 calories) lost a significantly higher percentage of bodyweight, 1.64% (95% CI 0.59-2.69) and 1.83% (95% CI 0.77-2.89), respectively. Women consuming 250 or more calories on Mondays than on weekend days lost a significantly lower percentage of bodyweight, including 1.38% less (95% CI −2.61 to −0.16) for those consuming 250 to 500 more on Mondays and 3.45% less (95% CI −5.29 to −1.61) for those consuming at least 500 calories more on Mondays. Compared with the men consuming more than 500 calories more on weekend days than on Mondays, there was no significant difference in percentage of bodyweight lost until caloric intake on Mondays was at least 50 calories higher than on weekend days: 50 to 250 calories more on Mondays −1.17%, 95% CI −2.11 to −0.24), 250 to 500 calories more on Mondays (−2.52%, 95% CI −3.69 to −1.34), and at least 500 calories more on Mondays (-3.37%, 95% CI −5.24 to −1.50).

In contrast to unadjusted analyses (see Table 3), there were no significant differences (*P*<.05) between women and men in the associations between factors and percentage of weight lost. In general, the patterns of association between these predictors and weight lost were similar for men and women. Only in the BMI category, the coefficients of the joint test were significantly different for women and men, with initial BMI appearing to affect men greater that it affected women.

**Table 2:** Unadjusted linear regressions of percentage of bodyweight lost among women and men who used the *Lose It!* mobile app (sex-specific intercepts for models are not reported; N=7007). Estimates in italics are significant at *P*<.05. BMI: body mass index. Ref: reference category.

Characteristics		Women			Men		
		Regression coefficient	Standard error	*P* value	Regression coefficient	Standard error	*P* value
**Age (years)^a^**							
	18-24	Ref			Ref		
	25-34	*.75*	*0.25*	*.003*	.41	0.41	.32
	35-44	*.65*	*0.25*	*.009*	.20	0.40	.62
	45-54	*1.04*	*0.25*	*<.001*	−.05	0.41	.91
	55-64	*.85*	*0.28*	*.003*	.04	0.43	.93
	65-80	−.03	0.37	.93	−*1.03*	*0.51*	*.05*
**BMI classification^a,b^**							
	Overweight	Ref			Ref		
	Obesity I	.34	0.17	.05	*1.27*	*0.24*	*<.001*
	Obesity II	*.84*	*0.22*	*<.001*	*1.16*	*0.32*	*<.001*
	Extreme obesity	*.82*	*0.24*	*.001*	*1.49*	*0.36*	*<.001*
**Weight loss plan (lb/week)^a^**							
	<1	Ref			Ref		
	1 to <1.5	*1.11*	*0.42*	*.008*	.12	0.56	.82
	1.5 to <2	*1.36*	*0.42*	*.001*	.51	0.56	.36
	2 to <4	*2.33*	*0.41*	*<.001*	*2.04*	*0.54*	*<.001*
**Daily calories on Mondays versus weekend days^a^**							
	More than 500 calories on weekend days	Ref			Ref		
	More than 250 to 500 calories on weekend days	.39	0.58	.50	−.52	0.49	.28
	More than 50 to 250 calories on weekend days	*1.64*	*0.54*	*<.001*	.17	0.45	.71
	More than or less than 50 calories	*1.83*	*0.54*	*<.001*	.11	0.48	.82
	More than 50 to 250 calories on Mondays	.50	0.54	.36	−*1.17*	*0.48*	*.01*
	More than 250 to 500 calories on Mondays	−*1.38*	*0.63*	*0.03*	−*2.52*	*0.60*	*<.001*
	More than 500 calories on Mondays	−*3.45*	*0.94*	*<.001*	−*3.37*	*0.95*	*<.001*

^a^Factor significantly associated (*P*<.001) with percentage of weight loss for that sex.

^b^Joint test comparing matching coefficients for women and men for a given factor is statistically significant (*P*<.001).

**Table 3:** Adjusted linear regression of percentage of bodyweight lost among women and men using the *Lose It!* mobile app (N=7007). Estimates in italics are significant at *P*<.05. BMI: body mass index. Ref: reference category.

Characteristics		Women			Men		
		Regression coefficient	Standard error	*P* value	Regression coefficient	Standard error	*P* value
**Age (years)^a^**							
	18-24	Ref			Ref		
	25-34	*.73*	*0.25*	*.003*	.15	0.40	.71
	35-44	.48	0.25	.05	.02	0.39	.95
	45-54	*.82*	*0.26*	*.001*	−.31	0.41	.44
	55-64	*.70*	*0.28*	*.01*	−.26	0.43	.55
	65-80	−.08	0.37	.84	1*1.04*	*0.51*	*.04*
**BMI classification^a^**							
	Overweight	Ref			Ref		
	Obesity I	.20	0.17	.24	*1.03*	*0.24*	*.001*
	Obesity II	*.44*	*0.22*	*.05*	*0.76*	*0.32*	*.02*
	Extreme obesity	.42	0.25	.09	*.93*	*0.37*	*.01*
**Weight loss plan (lb/week)^a^**							
	<1	Ref			Ref		
	1 to <1.5	*.89*	*0.42*	*.03*	−.18	0.56	.75
	1.5 to <2	*1.11*	*0.42*	*.01*	0.09	0.56	.87
	2 to <4	*2.07*	*0.42*	*<.001*	*1.37*	*0.55*	*.01*
**Daily calories on Mondays versus weekend days^a^**							
	More than 500 calories on weekend days	Ref			Ref		
	More than 250 to 500 calories on weekend days	.46	0.57	.42	−.34	0.48	.48
	More than 50 to 250 calories on weekend days	*1.64*	*0.53*	*.002*	.48	0.45	.29
	More than or less than 50 calories	*1.82*	*0.54*	*.001*	.42	0.48	.39
	More than 50 to 250 calories on Mondays	.54	0.54	.32	−.87	0.47	.07
	More than 250 to 500 calories on Mondays	−*1.35*	*0.62*	*.03*	−*2.27*	*0.60*	*<.001*
	More than 500 calories on Mondays	−*3.58*	*0.93*	*<.001*	−*3.42*	*0.94*	*<.001*
Intercept		*3.83*	*0.46*	*<.001*	*6.02*	*0.65*	*<.001*

^a^Factor significantly associated (*P*<.05) with percentage of weight lost for that sex.

Table 3 presents the coefficient estimates from the adjusted linear regression models, which included all four predictors and the joint tests comparing the coefficients for each factor between women and men, which revealed no significant differences. However, each factor was significantly associated with percentage of weight lost for women, men, or both. Age was significantly associated with weight loss for women but not for men, and the initial BMI category was significantly associated with percentage of weight lost for men, but not for women. For both women and men, the aggressiveness of weight loss plan and the caloric intake on Mondays versus weekend days were associated with percentage of weight lost.

For women, the patterns of association for the categories of caloric intake on Mondays versus weekend days were very similar in the adjusted and unadjusted analyses, with negligible difference in the magnitude of the coefficients. In the adjusted analyses, as compared with the women consuming 500 or more calories more on weekend days than Mondays, those consuming 50 to 250 more calories on weekend days or a similar amount of calories on both weekend days and Mondays (±50 calories) lost more body weight, with regression coefficients of 1.64 (95% CI 0.60-2.68) and 1.82 (95% CI 0.77 to 2.87), respectively; those consuming 250 to 500 calories and more than 500 calories more on Mondays than weekend days lost lesser bodyweight, with regression coefficients of −1.53 (95% CI −2.56 to −0.14) and −3.58 (95% CI −5.40 to −1.76) respectively.

For men, the magnitude of the coefficients for the associations between caloric imbalance and weight loss were also of similar magnitudes in the unadjusted and adjusted analyses. In the adjusted analyses, compared with men consuming 500 or more calories more on weekend days than Mondays, those consuming 250 to 500 calories and more than 500 calories more on Mondays than weekend days lost lesser bodyweight, with regression coefficients of -2.27 (95% CI −3.44 to −1.10) and −3.42 (95% CI −5.27 to −1.58), which are similar to the estimates in the unadjusted analyses. In contrast to the unadjusted analyses, percentage of bodyweight lost among those consuming more than 50 to 250 calories on Mondays than weekend days was no longer significantly different from those consuming at least 500 calories more on weekend days than Mondays.

## Discussion

### Principal Findings

In this sample of mobile phone app users for consistent weight loss, the lowest mean caloric intake was reported on Mondays and the highest was reported on Saturdays for both men and women. The results of this study indicate that consuming a consistent amount of calories per day throughout the week, or consuming slightly fewer calories per day on Mondays versus weekend days, are the most beneficial weekly patterns of caloric intake for weight loss. Consuming considerably more calories on weekend days versus Mondays was associated with less weight loss for women, but this association was weak for men. Conversely, consuming more calories per day on Mondays than on the weekends has negative implications for weight loss that increase with the magnitude of the difference in calories consumed. This negative association is particularly strong for women, with consuming 500 or more calories more on Mondays versus weekend days associated with at least 3% less bodyweight lost. For men, consuming 250 or more calories on Mondays versus weekend days was associated with 2.3% to 3.4% less bodyweight lost. These associations are also independent of the other variables examined. In particular, the initial BMI category and the aggressiveness of a weight loss plan had little effect on the observed associations between calories consumed on Mondays versus weekend days and percentage of bodyweight lost. Furthermore, the associations with percentage of weight lost were of greater magnitude for weekly patterns of intake than for other categories including age, initial BMI, and weight loss plan.

Potential mechanisms for this association have not been established. Possible explanations include associations between imbalanced caloric intake across the week and greater overall intake, macronutrient imbalance, energy expenditure patterns, or metabolism. Differences between weekday and weekend eating patterns may reflect differential eating triggers, such as eating out versus eating self-prepared meals, or differences between weekday and weekend lifestyle routines. Consistency in intake across the week may reflect higher self-monitoring or awareness of intake.

Caloric intake on Monday and the 3 consecutive weekdays were correlated (data not presented), and it is possible that moderated intake on Monday may set the tone for these subsequent weekdays. Therefore, Mondays might serve as an important reset point for the upcoming week.

### Limitations

This study has several limitations. First, caloric intake and bodyweight measurements are largely based on self-reporting. Some individuals may have used “smart” scales that connect to the *Lose It!* app, but we did not explore this. Most users did not record calories on a daily basis, and if days with missing data had systematically higher or lower consumption, this would have introduced reporting bias. Second, our analyses do not reflect caloric expenditure through physical activity. Although some users reported physical activity, this was not consistently recorded. Third, we do not know whether the observed associations would hold true for other *Lose It!* users or for dieters who do not use this app. The study sample consisted of consistent users, and different patterns of consumption and weight loss may occur for those with sporadic use, with short-term use, or who do not use the app at all. Fourth, although caloric balance may have a causal effect on weight loss, the observed association may be due to unmeasured confounding variables. For example, consistent caloric intake on Mondays versus weekend days may be associated with individuals having more consistent routines in general, such as regular exercise.

Additional research on the accuracy of self-reporting of caloric data through apps would be beneficial for research in this burgeoning area. With respect to the Monday effect, examining the associations among weekly variations, the diet compositions, and the weight loss outcomes may provide insights into potentially important and modifiable behaviors. As weekly variation was found to be an important predictor of weight loss, understanding factors that shape such a variation may provide another important avenue for promoting weight loss.

### Conclusions

The results of this study present an understanding of eating behaviors and obesity by explicating weekly patterns of caloric consumption and their association for successful weight control. By using mHealth in this way, we also gain a preliminary understanding of how engagement and effectiveness correspond with the users’ goals and the days of week. The size of the dataset contributes to the novelty and applicability of the study, and the study findings are valuable for obesity prevention because they further elucidate human behavior patterns with respect to weight loss and caloric decision-making. By increasing our understanding of these weight-determining factors, we can create relevant interventions and prevention schemes. The results of this study indicate the importance of weekly patterns of caloric intake for successful weight loss. Subsequently, diet and weight-related apps and interventions, such as *Lose It!,* can be designed and promoted. Ongoing prompts to engage or motivate users on Mondays may be well received among individuals who tend to demonstrate increased compliance to their diet plans following less-restricted eating habits on weekends. Similarly, a Monday reminder in the app may serve to retain lapsed users by appealing to their interest in health early in the week. Our analysis indicates that greater caloric intake on Mondays relative to weekend days is associated with poorer weight-loss outcomes, and that this pattern demonstrates a dose-response relationship. By reminding users of their goal each Monday, it may also be possible to limit exposure to this potentially damaging pattern among users who have previously shown signs of increased caloric intake at the beginning of the week.

## Abbreviations

BMI: body mass index
